# Supplementary material for: Spectroscopic Analyses Highlight Plant Biostimulant Effects of Baker’s Yeast Vinasse and Selenium on Cabbage through Foliar Fertilization
Source: Plants (Basel). 2023 Aug 21;12(16):3016. doi: 10.3390/plants12163016 (PMC10458166; doi:10.3390/plants12163016)
Supplement: Supplementary file 1 [file plants-12-03016-s001.zip › plants-2497954-supplementary.pdf]

Supplementary Materials

ATR-FTIR spectroscopy of cellulose, pectin, and lignin

For a better understanding of the FTIR spectra of cabbage samples and the effect of Se-VF, control spectra of MCC, Pct, andLgn were recorded and overlaid in Figure S1 and we give here a detailed characterization of the bands.

MCC has a peak maximum at 3331 cm^{-1} and a shoulder at 3279 cm^{-1} assigned to cellulose Iβ, the Iα allomorph being metastable [164,167] and sensitive in the hydrolytic obtaining of MCC. These assignments correlate with the prevalence of Iα towards Iβ in bacterial cellulose and some algae compared with higher plants [163–165], and also with the FTIR theory in which strong bonds or structures (Iβ>Iα) vibrate at higher frequencies (wavenumbers). Compared to MCC, Lgn has a broad absorption band due to its polyphenolic structure and a peak of reduced intensity at 3362 cm^{-1} in the hydrogen bonds region, which suggests, along with other spectral particularities later mentioned, that the Kraft lignin used in this analysis is predominantly a softwood type, the hardwood lignin spectrum being more sharp in this region with a peak around 3421 cm^{-1} [186]. Pectin (Pct) has more hydroxyl groups than Lgn but less than MCC, occurring from the galactose hydroxyls and galacturonic acid carboxyl moieties [78,118,168,176].

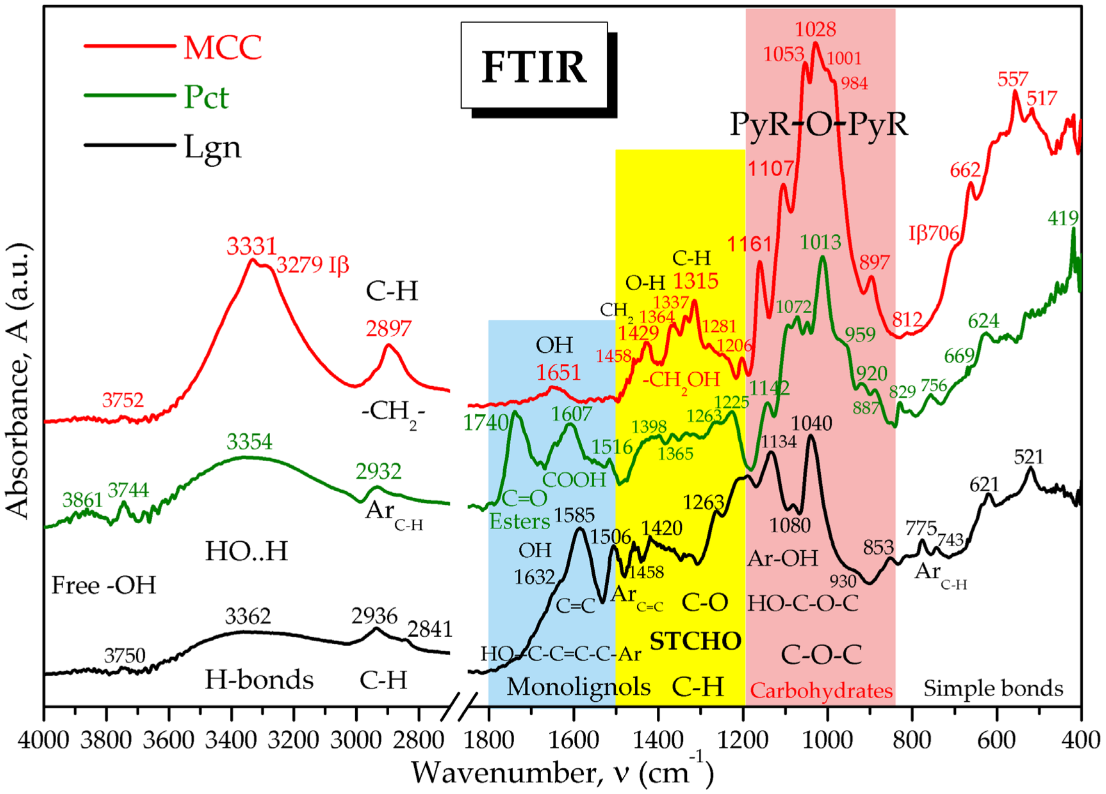

Figure S1. FTIR spectra of microcrystalline cellulose (MCC), pectin (Pct) and lignin (Lgn).

Table 3000. cm^{-1} is generally assigned to the C-H stretching vibrations coupled with adjacent functional groups [78,118,128,131,159,161,168,176,]. Pectin has a weak and broad C-H band with a peak at 2932 cm^{-1} (or 2939 cm^{-1}[168,176]), band assigned to aromatic C-H bonds and chelate OH groups. Lignin C-H band has the lowest intensity, while the peaks at 2936 and 2841 cm^{-1} suggest the asymmetric, respectively symmetric stretching vibrations of -C-H and -CH_2- in unsaturated alcohol moieties and aromatic rings of monolignols (guaiacyl, coniferyl, syringyl alcohols) [128,187].

In the first diagnostic region 4000-3000 cm^{-1}, characteristic to free and hydrogen-bound -OH or $-NH_2$, MCC has a peak maximum at 3331 cm^{-1} and a shoulder at 3279 cm^{-1} assigned to cellulose Iβ, the Iα allomorph being metastable [164,167] and sensitive to the hydrolytic process of obtaining MCC.These assignments correlate with the prevalence of Iα towards Iβ in bacterial cellulose and some algae compared with higher plants [163–165], and also with the FTIR theory in which strong bonds or structures (Iβ>Iα) vibrate at higher frequencies (wavenumbers). Compared to MCC, Lgn has a broad absorption band due to its polyphenolic structure and a peak of reduced intensity at 3362 cm^{-1} in the hydrogen bonds region, which suggests, along with other spectral particularities later mentioned, that the Kraft lignin used in this analysis is predominantly a softwood type, the hardwood lignin spectrum being more sharp in this region with a peak around 3421 cm^{-1}[186]. Pectin (Pct) has more hydroxyl groups than Lgn but less than MCC, occurring from the galactose hydroxyls and galacturonic acid carboxyl moieties [78,118,168,176].

The last diagnostic region (1800-1500 cm^{-1}), sometimes considered the first fingerprint region, can be seen as the shrinking mirror of the 3600-3000 cm^{-1} region at half wavenumbers, also specific for -O-H and for hydrogen bonds with crystallization water [161,188]. In this region MCC has one defined peak at 1651 cm^{-1} corresponding to -OH involved in inter- and intramolecular H-bonds and with crystallization water. Pct has a strong absorption band at 1740±50 cm^{-1} assigned to C=O vibration in esterified carbonyl COOR [78,93,118,119,159,168], being a reference band for the esterification degree of pectins in comparison with the free carboxyl groups -COOH around 1630-1645 cm^{-1}[118,119,121]. Lgn has a strong absorption band at 1585 cm^{-1} corresponding to aromatic ring vibrations [186], more exactly to conjugated phenols [128], and a front shoulder around 1632 cm^{-1} corresponding to aliphatic C=C stretching vibration combined with C-OH like in α,β-unsaturated alcohols, this band describing very well the structural vibrations of monolignols like *p*-coumaryl, coniferyl and synapyl alcohols. The next band at 1506 cm^{-1} is specific for Lgn since it corresponds to aromatic C=C ring vibration ($Ar_{C=C}$) [128,186].

The fingerprint region 1500-400 cm^{-1} contains "a forest of signals" specific to different vibration-rotation movements of intra-molecular bonds and functional groups induced by medium and low infrared energies, and it is generally recognized as very difficult, for complex samples, to be fully described since for a molecule with N atoms are theoretically possible 3N-6 fundamental vibrations [93]. At a first look, clearly distinguishable for MCC, the fingerprint region seems to be divided in three groups of signals: the first group of bands, between 1500-1200 cm^{-1}, corresponds to C-H bending vibrations in gluco-pyranosic rings (PyR) and scissoring of lateral $-C^6H_2$-OH [177], coupled with O-H bending. The second group of absorption bands, 1200-850 cm^{-1} is named the polysaccharides band and corresponds to various C-O bonds like C-OH, C-O-C pyranosic, respectively C-O-C glycosidic bond. The third and last group, between 850-400 cm^{-1}, corresponds to rocking and wagging vibrations of single bonds.

The pioneering FTIR study of Tsuboi in 1957 [161] on the fingerprint region of cellulose identified nine bands in the absorption region 1500-1200 cm^{-1}, out of which four were polarized parallel (1426, 1365, 1275 and 1232 cm^{-1}) and five perpendicular (1446, 1335, 1310, 1249 and 1204 cm^{-1}) to the fiber axis. Further deuteration of amorphous cellulose with D_2O and NaOD evidenced that the bands at 1446, 1335, 1232 and 1204 cm^{-1} are influenced by hydrogen-bonding with D_2O, therefore assigned to -OH in-plane deformation, while the bands at 1426, 1365, 1310, 1275 and 1249 cm^{-1} are not affected by deuteration, therefore assigned to C-H deformation vibrations. In the polysaccharides region 1200-850 cm^{-1}, Tsuboi [161] identified a number of ten absorption bands, respectively: 1161, 1117, 1106, 1078, 1069, 1030, 1006, 988, 965, and 895 cm^{-1}, all ten showing parallel dichroism and grouped as C-O, C-C and CH_2 stretching and rocking vibrations. Although it is beyond the scope of the present study to exactly assign each absorption band to the exact type of atomic vibration-rotation due to the increasing difficulty with the complexity of the molecular system, particular vibrations are attempted in Table A1 by structural and spectral comparison between microcrystalline cellulose (MCC), pectin (Pct), and lignin (Lgn), while referring also to relevant studies.

Table S1. Assignment of FTIR absorption bands of MCC, Pct, Lgn and cabbage control Co.

**Bond/group**	**Wavenumbers (ν), cm^{-1}**, for: **Co**-cabbage control outer leaves, **MCC**-microcrystalline cellulose, **Pct**-pectin, **Lgn**-lignin.				**Observations / References** (main tools [93,128,131,158,159,161,188])
	**Co**	**MCC**	**Pct**	**Lgn**	PyR - pyran ring; Ar - aromatic ring; @ - around.
Free -OH	3838±60<br>3740±40	3752±40	3861±100<br>3744±40	3750±40	-unassociated, free hydroxyl groups @3700 cm^{-1} (@2.7μm [158]);
-H bonds with -OH and/or -NH<br>-Ar-H and/or C-N..H @3020 cm^{-1}	3448±20<br>3281±200<br>3227±10<br>3082±20<br>3018±30	s3331±250<br>3289 Iβ	m3354±250	w3362±250<br>sh3022±5	-between 3600-3400 cm^{-1} might be assigned the single H-bonds, while between 3400-3000 cm^{-1} might prevail the double H-bonds [160];<br>- Iα @3240 cm^{-1}, Iβ @3275 cm^{-1}[146,163,164];
- C-H coupled with -OH in<br>$-CH_2$-OH	sh2960<br>vs2920±30	sh2968,<br>sh2943	w2932	w2936	-asym. νCH_2-OH at 2967 cm^{-1}[161] and H-bridge, in correlation with the region at half ν=1485-1455 cm^{-1}; -asym. $-CH_2-$ @2925 cm^{-1}[128]; - C-H @3.4 μm (@2941 cm^{-1}) [159];
- sym. C-H in $-CH_3$, $>CH_2$, C-H	s2851±25	s2897±70	w2857±30	w2841±30	-C-H in cellulose [130,161] ;<br>-sym. $-CH_2-$ @2855 cm^{-1}[128] or @2851 cm^{-1}[161];
- C=O in COOR @1740 cm^{-1} and in COOH @1730 cm^{-1}	s1738±50	-	s1740±50<br>sh1728±5	-	- C=O in esters @5.73 μm (1745 cm^{-1}) [158,159];<br>- C=O @1736 cm^{-1} in COOH [93];<br>- C=O in esterified carbonyl COOR in Pct [78,118,121,168];
-OH in PyR-OH, CH_2-OH and H-bonds with H_2O	sh1645±5	m1651±10	sh1645±5	sh1632±5	- @1645±5 cm^{-1} -OH involved in H-bonds with crystallization H_2O in polysaccharides [93];<br>- OH in C^6-OH @1639 cm^{-1} (@6.1μm) [159];
- C=O in aminoacids, esters, amide I, with amide II shoulders for - C-N-H	s1603±10<br>sh1574	-	s1607±30<br>sh1568<br>sh1551<br>sh1541	-	- C=O in COOH @1612 cm^{-1}[93], pectin carboxyls[118];<br>- C-N-H in amines and amides @1550 cm^{-1}, C-N in amide II @1530 cm^{-1}[126,128];
- $Ar_{C=C}$ coupled with C-H	-	-	-	s1585±50	- benzene ring in phenyl glycosides @1580 cm^{-1}[93];<br>-aromatic C=C @6.35μm (@1575cm^{-1})[158], asymmetric;
- $Ar_{C=C}$ coupled with C-H,<br>- Ar-C=O for pectins	sh1522	-	1516±10<br>Ar-C=O	s1506±25<br>sh1490<br>$Ar_{C=C}$	- aromatic C=C @6.65μm (@1504cm^{-1}) [158], symmetric;<br>- C=O in COOH for Pct;<br>- aromatic / heterocyclic νC-C @1490 cm^{-1}[93];
C-H coupled with >CH-OH, and correlated with the H-bridge @2xWN 2970-2920 cm^{-1}	-	sh1474<br>sh1458	-	m1458,<br>sh1448	- δC-H in saccharides [188] with a shoulder for, most probably, $-C^6H_2$-OH in MCC;<br>$-CH_2$-OH @1446 cm^{-1} evidenced by deuteration [161];<br>- C-H bending @6.9μm (@1449cm^{-1}), also named "crystalline band" [158];
C-H , $-CH_2-$	m1423±20	m1429±5	w1427±5	w1420±10	$-CH_2-$ @6.95μm, (@1439cm^{-1}) [158], CH_2 sym. bend @1426 cm^{-1} unaffected by deuteration [161];
C-H in $-CH_3$, $-CH_2-$,-C^6H_2-OH	s1367±10	m1364	w1365±10	w1364±10	- sym. parallel C-H @1365 cm^{-1} unaffected by deuteration [161]; $-CH_3$ in cellulose acetate @7.2μm (@1389 cm^{-1}) [159];
C-OH and Ar-OH	-	w1337	w1332	w1339	-C-OH @1335cm^{-1} evidenced by deuteration [161];
C-H	-	m1315	w1319	w1327	-C-H as. perpendicular, unaffected by deuteration [161];
C-H and O-H in -CH_2-OH	-	w1281<br>w1248	w1263	s1263	-OH in $-C^6H_2$-OH @7.8 μm (@1282 cm^{-1}) [159];<br>-C-H unaffected by deuteration @1275 cm^{-1} and @1249 cm^{-1}[161]; -CH-OH strong in lignin due to polyphenols;
$-CH_2$-OH<br>$-CH_2$-O-$CH_{(2)}$- - PyR-O-PyR	s1221±40	w1234	m1225	-	$-C^6H_2$-OH @1232 cm^{-1}[161];<br>-C-O-C- in ester bond [159];<br>- PyR-O-PyRglycosidic link / aryl ethers [93];
$-CH_2$-OH, $-C^6H_2$-OH	-	w1206±5	-	1200±30<br>sh1211<br>s1188	-bending of $-CH_2$-OH ($-C^6H_2$-OH @1204 cm^{-1}[161]), similar with trehalose, maltose and maltotriose[188];<br>-Ar-C-OH in monolignols - Lgn;

C-O-C coupled with C-OH / PyR-OH	sh1144	s1161	m1142	s1134	-PyR-O-PyRasymmetric stretching of glycosidic linkage in polysaccharides [189]; - C-O-C alkyl ethers [93]; - ring skeleton @8.6 μm (@1162 cm^{-1}) [159];
C-O-C cyclic	sh1092	s1107	m1094	-	- C-O-C cyclic in glucopyranosic ring (cyclic ethers [93]);
C-O-C aliphatic	sh1063	-	m1072	w1080	-alkyl ethers C-O-C [93];
C-O in C-OH	-	s1053	w1047	vs1040	- C-OH @1053 cm^{-1} (@9.5 μm) in alcohols [159];
PyR-O-PyR	-	vs1028	-	-	-symmetric stretching of PyR-O-PyR glycosidic;
C-OH / C-O	s1016	ah1001	vs1013	-	-symmetric C-O vibration in MCC and Pct;
PyR-O-PyR / C-O-C/	sh968	sh984	sh959	-	- glycosidic linkage in poly/oligo/saccharides [189], absent in monosaccharides and affected by acidic hydrolysis of oligosaccharides [188];
Ar-OH, COOH	-	-	w920	sh930	-COOH in Pct and Ar-OH in Lgn;
PyR-O-PyR	sh901	m897	w887 sh849	853	- glycosidic link [190,191] coupled with aromatic C-C(-O) or C-O-C inside PyR[192] ; -amorphous band @11 μm (@909cm^{-1}) and also -CH_2- @11.15 μm (@897cm^{-1}) [158];
PyR-C / C-C	sh831	w812	829, 806	816	- PyR-C in PyR-C-OH moiety for MCC;
Ar_{C-H}	-	-	756	w775 w743	-aromatic C-H @13μm (@769cm^{-1}) [158]; - aryl and pyranose ring breathing @751 cm^{-1} , Ar_{C-H} @755 cm^{-1}[93]; - γC-H @745-755 cm^{-1} in phenyl glycosides [93];
-CH_2-OH in Iβ	-	706-Iβ	-	-	- CH_2 rocking in Iβ @710 cm^{-1}[146]; - Iβ @715 cm^{-1} assigned to -CH_2-OH [193];
PyR H-C-C-H C-OH	m660	m662	w669	sh635	- C-C rocking @690 cm^{-1} in phenyl glycosides [93]; - C-O-H in cellulose perpendicular to plane [161]
C-H	m602	w608, 592	w624, 575	w621	- in-plane rocking vibrations of aliphatic C-H;
C-O	-	m557	w554	-	- in-plane rocking vibrations of C-O;
C-C, ArC-C, PyRC-C	w523	w517	w532	m521	- in-plane rocking of C-C, aromatic / PyR breathing;
C-C	s457	sh434	w457	w457	- C-C skeleton vibration / Ar ring vibration;
C-C-O	-	m419	m419	-	- C-O coupled with C-C in Pct and MCC.

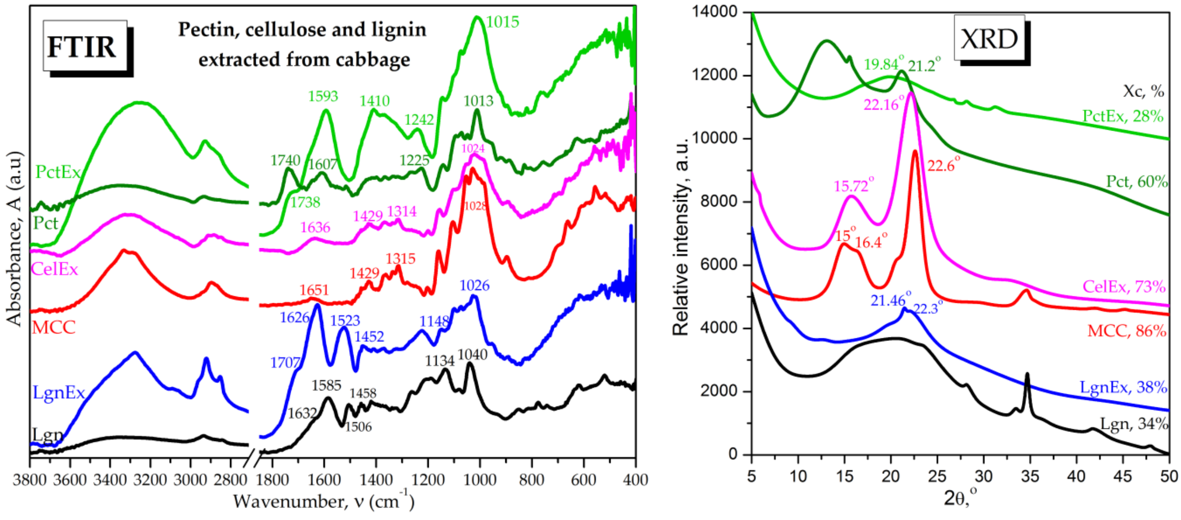

(a) (b)

Figure S2. (a) FTIR spectra and **(b)** XRD diffractograms of extracted cabbage samples, compared with commercial controls: PctEx - pectin extracted from cabbage; Pct - commercial pectin from citrus peels; CelEx - cellulose extracted from cabbage; MCC - commercial microcrystalline cellulose; LgnEx - lignin extracted from cabbage; Lgn - commercial alkali lignin.

The spectral similarities between extracted pectin, cellulose and lignin from untreated cabbage and the commercial biocompounds are visible both in the FTIR spectroscopy and X-ray diffraction

presented in Figure S4, but also small particular differences can be observed. The extracted cellulose from cabbage presents the highest similitude degree with MCC both in FTIR and XRD analyses. FTIR similarities between CelEx and MCC are visible in all absorption bands, starting with the hydrogen bonds and C-H vibrations in the diagnostic region, followed by -OH bands around 1640 cm^{-1}, the structural carbohydrates bands around 1429 and 1314 cm^{-1} and the polysaccharides bands between 1200-850 cm^{-1}. The two main diffraction peaks of CelEx at 15.72° and 22.16° are similar in intensity ratio between the two peaks with MCC, while the peak position suggests a higher cellulose Iα contribution in the cellulose from cabbage CelEx.

Extracted pectin PctEx shows FTIR spectral similarities with commercial Pct from citrus peels in the amide bands with peaks at 1738 and 1593 cm^{-1}, in comparison with the peaks at 1740 and 1607 cm^{-1}, although the intensities differ, the carboxylic band around 1593 cm^{-1} being more intense in cabbage pectin PctEx. Also, the main C-O vibration at 1013 cm^{-1} in Pct is similar with the one at 1015 cm^{-1} in PctEx, while the adjacent -OH bands around 1100 cm^{-1} together with the hydrogen band around 3300 cm^{-1} suggests more -OH groups in PctEx. The XRD patterns of Pct and PctEx are different, suggesting different crystallization patterns between the two pectin samples. The XRD of PctEx is in agreement with the XRD of the cabbage sample (Figure 3). Regarding the two lignin samples, the FTIR spectra suggests a more aliphatic nature for the lignin from cabbage LgnEx with the sharp C-H absorption bands around 2900 cm^{-1} and more C=O groups with the absorption band around 1707 cm^{-1}. The polyphenolic structural similarities between LgnEx and Lgn are suggested by the bands around 1626, 1523, 1452, 1148 and 1026 cm^{-1}. The XRD pattern of Lgn shows a broad diffraction pattern, and the LgnEx diffractogram is more centered around the 22° angle and suggests a more crystalline character, probably induced by the aliphatic chains observed in FTIR spectrum.

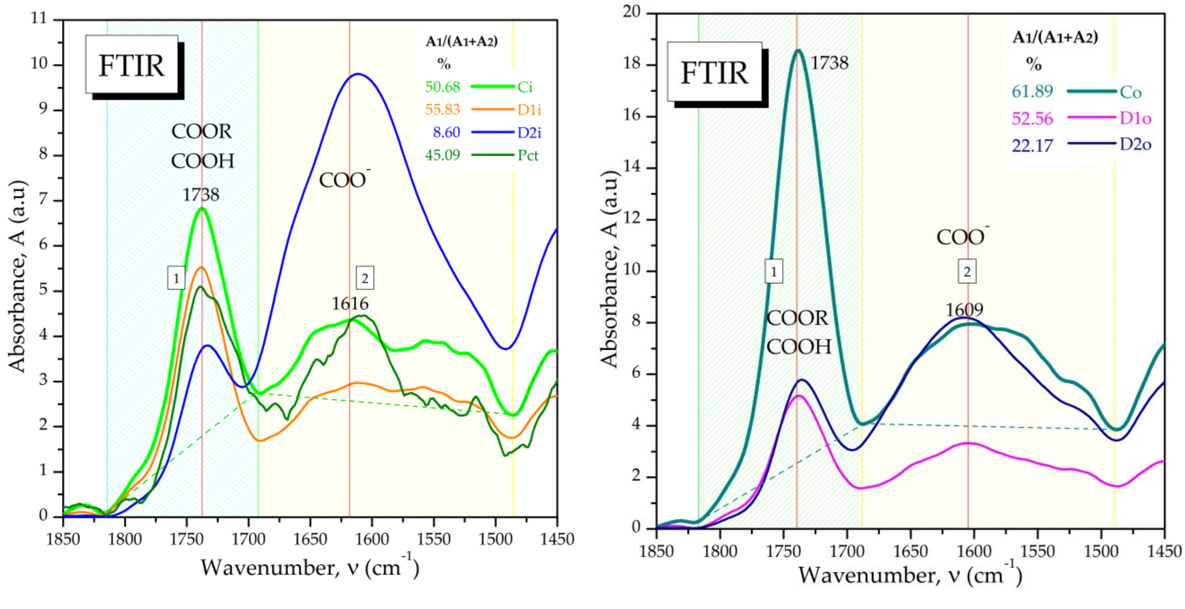

(**a**) (**b**)

Figure S3. Integration of FTIR absorption bands of COOR/COOH @1738 cm^{-1} and COO^{-} @1610 cm^{-1} and evaluation of the degree of methylesterification (DM, %) for: (**a**) Cabbage inner leaves and citrus pectin (Pct); (**b**) Cabbage outer leaves. Ci - control cabbage inner leaves; Co - control cabbage outer leaves;. D1i - dosage 1 (1 L/ha) on inner leaves; D1o - dosage 1 on outer leaves; D2i - dosage 2 (3 L/ha) on inner leaves; D2o- dosage 2 on outer leaves.

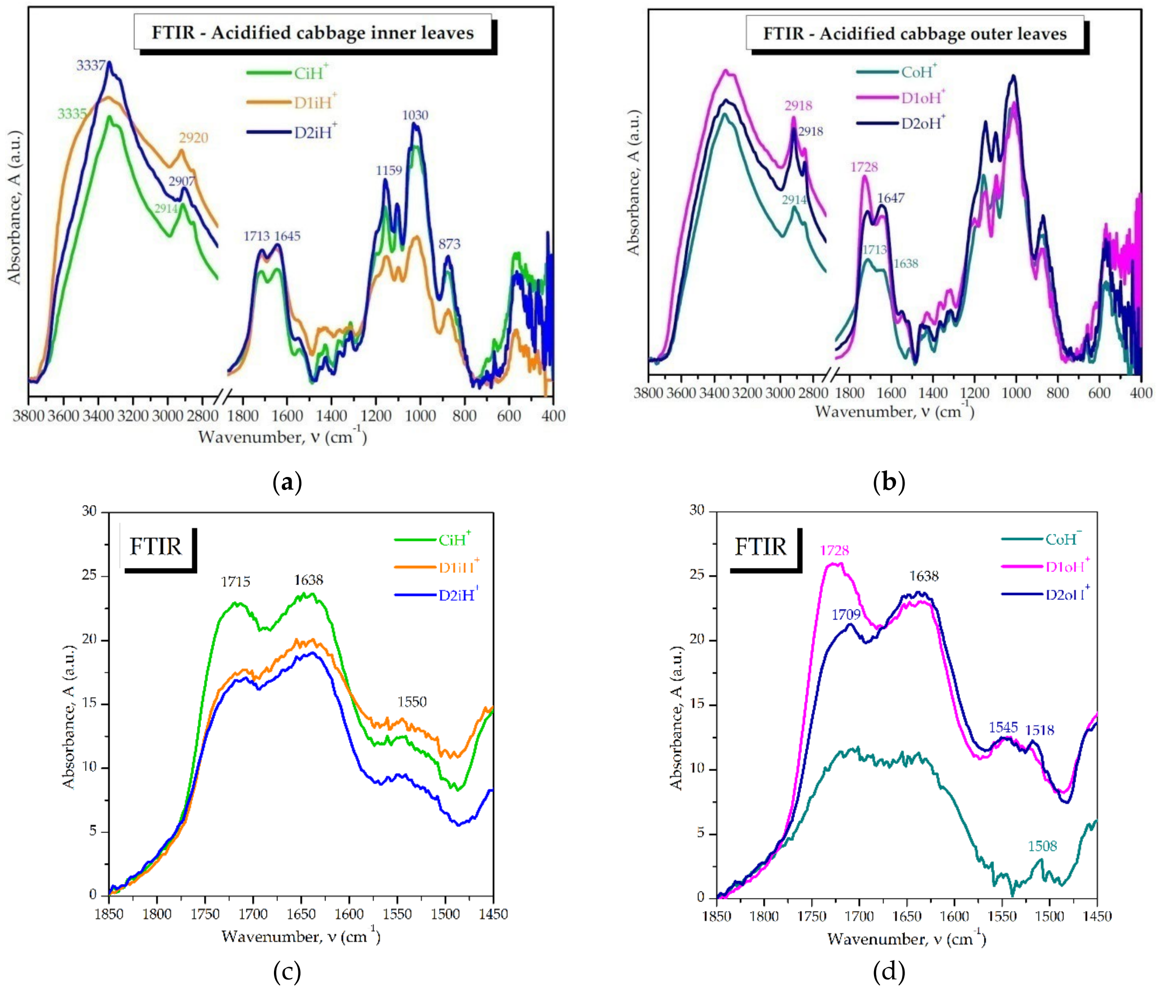

Figure S4. FTIR spectra of acidified cabbage samples: (**a**) Cabbage inner leaves; (**b**) Cabbage outer leaves; (c, d) Close view of the 1850-1450 cm^{-1} FTIR region of inner leaves (c) and outer leaves (d). Ci - control cabbage inner leaves; Co - control cabbage outer leaves; D1i - dosage 1 (1 L/ha) on inner leaves; D1o - dosage 1 on outer leaves; D2i - dosage 2 (3 L/ha) on inner leaves; D2o- dosage 2 on outer leaves.

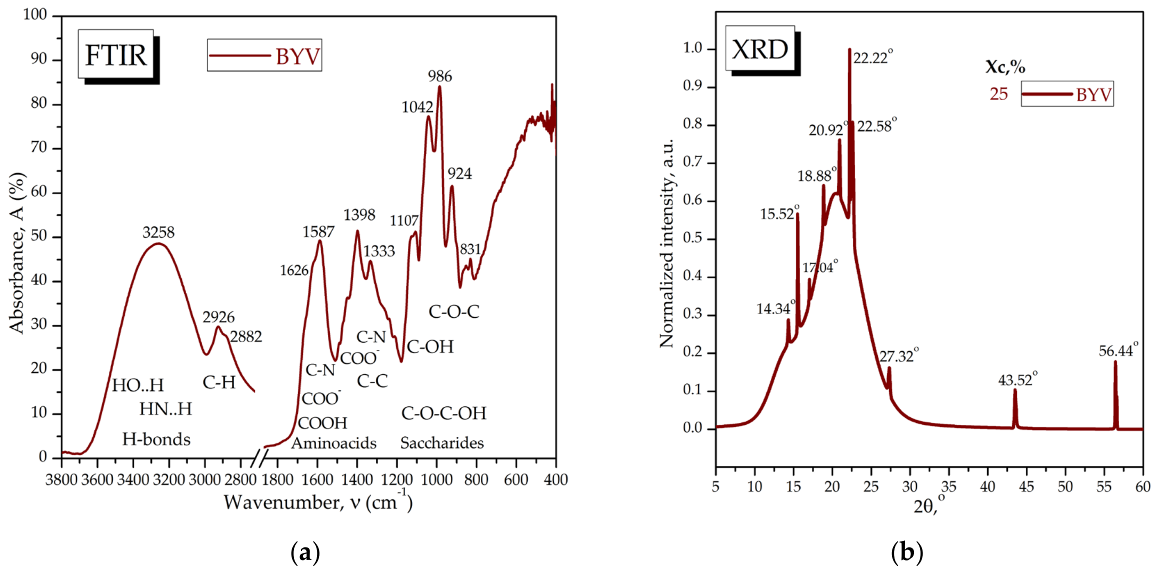

Figure S5. Characterization of baker's yeast vinasse: (**a**) FTIR spectrum; (**b**) X-ray diffractogram.

FTIR spectrum of BYV presented in Figure S5(a) is characterized in the diagnostic region by strong hydrogen bonds centered around 3258 cm^{-1} originating from -OH, -COOH and -NH- groups, respectively C-H bonds vibration at 2926 and 2882 cm^{-1} belonging to methyl groups in glycine betaine, aminoacids, sugars and other biocompounds. In the fingerprint region are differentiated the aminoacids band with a peak at 1587 cm-1 for C-N bond and carboxyl ions, respectively a shoulder at 1626 cm^{-1} for -COOH. The structural carbohydrates band in the region 1500-1200 cm^{-1} presents two absorption peaks at 1398 and 1333 cm^{-1}, assigned to carboxyl ions and C-N bonds vibration coupled with structural C-C bonds. BYV content of saccharides is depicted by the absorption band between 1200-800 cm^{-1}, with absorption peaks at 1107, 1042, 986, 924 and 831 cm^{-1} assigned to C-OH and C-O-C bonds vibration in sugars.

The XRD analysis presented in Figure S5(b) is evidencing a predominant amorphous character with a crystallinity degree of 25% with additional diffraction peaks characteristic for glycine betaine, glucose, fructose, and their combinations with acids and aminoacids like: alanyl glycine, allylglycine, betaine ascorbic acid, betaine citrate, betaine malonic acid, betaine oxalate, betaine succinic acid, betaine tartrate, carbonyl glycine, deoxyfructopyranosyl glycine, fructosazone, glucoseurea, glutaric acid glycine, glycine phtalic acid, glycine tartaric acid, glycyl-prolyl-leucyl-glycine, palmitoglycine, serylglycine, uracyl-carboxyethyl-glycine.
